# Knowledge, experiences and attitudes of dental and health care personnel in Sweden towards infant dental enucleation

**DOI:** 10.1007/s40368-018-0351-y

**Published:** 2018-07-09

**Authors:** J. Barzangi, L. Unell, K. Skovdahl, K. Arnrup

**Affiliations:** 1 Dental Research Department, Public Dental Health Service, Örebro, Region Örebro County Sweden; 2 Specialisttandvårdskliniken, Västmanland Hospital Västerås, Västerås, Region Västmanland County Sweden; 3 School of Health Sciences, Örebro University, Örebro, Sweden; 4 Faculty for Health and Social Sciences, University of South-Eastern Norway, Drammen, Norway

**Keywords:** Traditional medicine, Africa, Dental staff, Medical staff, Surveys and questionnaires, Emigrants and immigrants

## Abstract

**Purpose:**

To examine self-rated knowledge of clinical experiences and attitudes towards the practice of infant dental enucleation among dental and health care personnel in Sweden.

**Methods:**

A questionnaire survey was performed among 776 licensed dental and health care personnel working in emergency departments, midwifery, child health centres, school health services and public dental health services in 10 Swedish cities. The response rate was 56.2% (n = 436).

**Results:**

Fewer than a fifth of the respondents reported self-rated knowledge of the practice. Approximately 13% of personnel encountering children professionally believed they had seen subjected patients in their clinical practice. Personnel with self-rated knowledge and clinical experience worked mostly in dental care. Additionally, the personnel had diverging attitudes regarding agreement and disagreement concerning professional responsibility for patients subjected to or at risk of infant dental enucleation.

**Conclusions:**

The study indicated there is need for increased knowledge about the practice and for clarification of obligatory responsibilities among dental and health care personnel regarding management and prevention of cases of infant dental enucleation.

## Introduction

Infant dental enucleation (IDE) is a practice comprising removal of tooth buds in children mainly below the age of 1 year (Welbury et al. 1993; Asefa et al. 1998; Accorsi et al. 2003). The general purpose is to treat or to prevent symptoms and diseases such as diarrhoea and fever (Baba and Kay 1989; Mutai et al. 2010). IDE is practised mainly in areas of the Eastern African countries Ethiopia, Kenya, Somalia, Sudan (and nowadays also South Sudan), Tanzania and Uganda (A/Wahab 1987; Hiza and Kikwilu 1992; Ahmed et al. 1994; Asefa et al. 1998; Accorsi et al. 2003; Food Security and Nutrition Analysis Unit 2007; Mutai et al. 2010). Prevalence from a few per cent to 100% has been reported in different areas and study populations (A/Wahab 1987; Baba and Kay 1989; Hiza and Kikwilu 1992; Kikwilu and Hiza 1997; Accorsi et al. 2003; Wanzala et al. 2008; Kipchumba 2012; Tirwomwe et al. 2013; Gebrekirstos et al. 2014). Furthermore, prevalence has been indicated among children of residents of Eastern African origin in England (Rodd and Davidson 2000) and Israel (Holan and Mamber 1994; Davidovich et al. 2013). These latter studies have also suggested the practice may be continuing among Eastern African immigrants.

Most commonly, the primary canine buds in the mandible are enucleated (Baba and Kay 1989; Hiza and Kikwilu 1992), and both boys and girls appear to be equally subjected (Welbury et al. 1993; Rodd and Davidson 2000). The enucleations are performed by local health practitioners or older family members using simple non-sterile instruments such as knives, chisels or fingernails. Also, no pain relief is given in advance to the infants (Baba and Kay 1989; Accorsi et al. 2003; Tirwomwe et al. 2013; Teshome et al. 2016). Consequently, IDE has been suggested to cause complications both for general health and in the oral area. Examples of general health complications are excessive bleeding and systemic infections leading to septicaemia or meningitis deaths due to general health complications have also been reported (Iriso et al. 2000; Accorsi et al. 2003). The oral complications have been seen to impact both primary and permanent canines, and sometimes adjacent teeth. These comprise local infections, absence or impaction of teeth, eruption deviances and dental hard tissue defects or disfigurations (Matee and van Palenstein Helderman 1991; Welbury et al. 1993; Rodd and Davidson 2000; de Beavis et al. 2011; Noman et al. 2015). Impact on occlusion has been indicated as well (Holan and Mamber 1994; Hassanali and Odhiambo 2000).

Between 2010 and 2015, Sweden has had an increase, from 95,000 to at least 160,000, in the population of residents who either were born in, or whose parents were both born in, Eastern Africa (Statistics Sweden 2016). The presence in Swedish dental care of children who have possibly been subjected to IDE has been reported in a previous study (Barzangi et al. 2014). Indications of both prevalence and continuance of IDE among immigrants in Europe pose new challenges for Swedish dental and health care (Holan et al. 1994; Rodd and Davidson 2000; Davidovich et al. 2013; Holder 2016). These challenges may include awareness and basic knowledge of IDE, and responsibilities regarding management of subjected cases and prevention of continuance of the practice. Dentaid, a dental aid organisation based in UK, has made efforts to raise awareness of IDE in the public and among dental professionals in Europe as well as in East Africa (Longhurst 2010a, b; Gollings 2011, 2017). As of now, no study has been found on basic knowledge of and attitudes towards management of IDE cases among dental and health care personnel in Sweden. Also, no initiatives to raise awareness in that context are known. Thus, the aim was to examine self-rated basic knowledge on, clinical experiences of and attitudes towards the practice of IDE among dental and health care personnel in Sweden.

## Materials and methods

### Design and setting

The study was conducted as a questionnaire survey set in four Swedish counties. Population statistics for all municipalities in the counties in 2013 were obtained from the administrative agency Statistics Sweden regarding numbers of residents and their countries of birth or, if born in Sweden, the countries of birth of both their parents. The percentage of residents born in, or having both parents born in, an Eastern African country was calculated for each municipality. The countries were included based on the UN definition of Eastern Africa (United Nations 2014). Sudan was also included, as IDE has been reported to be practised there (Ahmed et al. 1994).

The proportions of residents with Eastern African origin varied from 0 to 5%. After ranking from highest to lowest percentage, 10 municipalities of comparable population sizes were chosen: four with percentages of ≥ 3% (called high municipality group) and six with proportions of ≤ 1% (called low municipality group). The sample frame in each group of municipalities was identified regarding the clinical settings to be included in the study.

### Respondents and sampling

A purposive sampling method was applied. Eligible for the study were dental and health care personnel working in clinical settings in which they encountered children (with their parents/guardians), and/or parents-to-be, as patients. The study population was therefore defined as dental and health care personnel who might be first in their professions to encounter IDE in a time period during which, according to the literature,

- parents-to-be, or parents or guardians of children, may consider performing IDE;
- infants may be exposed to the practice, or enucleations may be detected; or
- acute or long-term complications may be manifested.

The majority (91–100%) of children in the selected counties were registered at the public dental health service for regular check-ups and treatments (Suslick 2014). Therefore, licensed dental hygienists and dentists in all public dental health service clinics were invited to participate.

In health care, licensed doctors, nurses and midwives performing regular check-ups of parents-to-be during pregnancy (at midwifery centres), or of children after birth up to the age of 6 years (at child health centres) were invited to participate. Also invited were personnel working in emergency care and school health services.

Directors or equivalents of all the clinics (and centres) were contacted. Information was given about the study, and the clinics were invited to participate by distributing the questionnaire among eligible personnel. Information was obtained about the number of eligible personnel and their gender. A total of 118 clinics/subdivisions were contacted, of which 110 accepted and eight declined participation or did not respond. Of the eight clinics/centres that were not included, two provided dental care. Reasons for declining participation were as follows: great workload, study not considered relevant to their personnel or no reason given.

Questionnaires with return envelopes were sent to the clinics. Enclosed with all questionnaires was a cover letter containing information about the study and the voluntary and confidential nature of participation. If necessary, a reminder was made 3 weeks later with a request that the director or equivalent forward it to the personnel. The process was repeated, if necessary, an additional 4–5 weeks later.

### Questionnaire construction and data collection

Data were collected using a printed, newly constructed and self-administered semi-structured questionnaire. In addition to asking about demographic and professional characteristics, the questionnaire contained three substantive sections:

Section 1 concerned self-rated basic knowledge of IDE and the source of the knowledge. Accordingly, the respondents rated their familiarity with basic information about IDE. This section had a matrix question on basic knowledge that contained statements about IDE to be rated on a scale as follows: (1) *no knowledge*; (2) *very little knowledge*; (3) *some knowledge*; or (4) *good knowledge*. The source of the knowledge was obtained through a multiple response question with additional open-answer space.

Section 2 concerned the personnel’s direct clinical experiences of IDE. The questions contained yes/no or multiple-response alternatives and open-answer spaces about encountering clinical cases, either confirmed or being assessed immediately or in retrospect as having been subjected to IDE. Respondents could also describe complications assessed as having been caused by IDE.

Section 3 concerned attitudes towards IDE regarding professional responsibility for its detection, examination, treatment, referral and prevention in their professions. This section contained questions with statements to which respondents rated their responses on a five-point Likert scale: *fully agree, partly agree, don’t know, partly disagree, disagree*.

Content validity was assessed via consensus between the authors. Four dentists were asked to comment on the questions with regard to language, comprehension and relevance. The study was approved by the Regional Ethical Review Board in Uppsala, Sweden (reference number 2014:543).

### Statistical analysis

All descriptive and statistical analyses were performed using IBM© SPSS© Statistics version 22.0 (IBM Corp., Armonk, NY, USA). Grouping was made according to clinical setting: Personnel in public dental health services were grouped and labelled dental care; personnel in emergency departments, child health services, midwifery services and school health services were grouped and labelled health care. Open answers were quantified in categories based on similarities and incorporated into existing response alternatives or new alternatives. Significance tests were performed using Chi^2^ test. Dichotomies were made of response alternatives of statements on basic knowledge between *no knowledge* and all other options (called *any knowledge*). Peak value of basic knowledge was calculated as the highest level of response alternative (from 1, no knowledge, to 4, good knowledge) of four introductory statements:

*A: There is a traditional practice consisting of removing tooth buds in infants*,

*B: It is being practised in Eastern Africa (among the countries Ethiopia, Kenya, Somalia, Sudan, Tanzania and Uganda)*,

*C: It is the milk tooth buds of the lower jaw that are removed, and*

*D: It is done to treat bodily diseases (such as diarrhoea, fever or vomiting)*.

The statements were chosen because they were assessed to contain essential information that defined IDE. Trichotomies were made of response alternatives of statements on attitudes by combining *fully agree* with *partly agree*, and by combining *partly disagree* with *disagree*. Multivariate analysis was performed using binary logistic regression. For the purpose of regression analysis, peak value variable was used as data on knowledge, and was dichotomised between *no knowledge* and all other options (*any knowledge*). Concerning data on attitudes regarding responsibilities in multivariate analysis, *fully agree* and *partly agree* were combined, as were *I don’t know, partly disagree* and *disagree*. Significance level was set at *p* < 0.05.

## Results

### Sample characteristics

A total of 776 questionnaires were sent to eligible respondents, of which 451 were returned. Fifteen were excluded from analysis due to insufficient demographic data, leaving 436 respondents and an overall response rate of 56.2%. Of these, 80.3% were women. Distribution of respondents by gender, municipality group and clinical setting are presented in Table 1. The mean age was 46.9 years (SD 11.4, range 24–73), and mean working experience in the profession was 15.9 years (SD 11.3, range 1–45), both equal between genders. Also, 93.8% reported being clinically active with patient-related clinical activity of 20 or more hours a week. No differences were seen between the genders in any of the analyses, and gender is therefore not presented in the results.

**Table 1:** Distribution of respondents by gender, municipality group and clinical setting

	Questionnaires sent (n)	Responded (n)	Response rate (%)
Total	776	436	56.2
Gender			
Men	199	86	43.2
Women	577	350	60.6
Municipality group			
High	299	169	56.5
Low	477	267	56.0
Clinical setting			
Health care^a^	621	346	55.7
Dental care^b^	155	90	58.1

^a^Doctors, midwives and nurses

^b^Dental hygienists and dentists

### Basic knowledge, and sources of knowledge, of IDE

An overview of statements on and respondents’ self-rated basic knowledge of the practice is shown in Table 2. Of the respondents, 83.4% reported not having basic knowledge of any of the statements A–D (i.e. peak = 1), and 16.6% (n = 72) reported very little, some or good knowledge about at least one statement (peak > 1). More respondents in the high municipality group had knowledge of tooth buds being removed as a traditional practice, the practice being done to treat bodily diseases and presence of subjected patients in Europe/Sweden. Additionally, there were differences between clinical settings regarding all statements; more personnel in dental care had knowledge than did personnel in health care. Association analysis between self-rated knowledge as dependent variable, and municipality group and clinical setting as independent variables, showed that respondents in the high municipality group were more likely to report self-rated knowledge (OR 2.22, CI 95% 1.15–4.28, *P* = < 0.05) than were respondents working in dental care (OR 31.01, CI 95% 16.03–59.99, *P* = < 0.001).

**Table 2:** Respondents’ self-rated basic knowledge of infant dental enucleation (IDE) by total respondents and response statement alternatives, and by municipality group and clinical setting

Statements	Total		Municipality group^a^			Clinical setting^a^		
Question: To what extent do you have previous knowledge of the following	No knowledge (%)	Any knowledge (%)	High (%)	Low (%)	*P*	Health care (%)	Dental care (%)	*P*
	429–433^b^		164–167^b^	264–266^b^		326–332^b^	89–90^b^	
A: There is a traditional practice consisting of removing tooth buds in infants	84.1	15.9	21.0	12.8	*	5.0	57.8	***
B: It is being practised in Eastern Africa (among the countries Ethiopia, Kenya, Somalia, Sudan, Tanzania and Uganda)	87.3	12.7	16.3	10.5	NS	3.2	48.9	***
C: It is the milk tooth buds of the lower jaw that are removed	86.5	13.5	16.3	11.7	NS	2.6	54.4	***
D: It is done to treat bodily diseases (such as diarrhoea, fever or vomiting)	87.2	12.8	17.0	10.2	*	2.4	52.2	***
E: The practice can cause bodily complications (such as bleedings and infections in the body)	74.5	25.5	26.9	24.5	NS	16.8	58.9	***
F: The practice can cause dental complications in the removal area (such as missing teeth, teeth being misshaped or erupting incorrectly)	71.8	28.2	30.5	26.8	NS	15.9	75.3	***
G: There are patients of Eastern African origin in Europe, including Sweden, that have been subjected to it	14.2	85.8	19.9	10.6	**	3.8	53.3	***
H: Studies show the practice may be continuing among people of Eastern African origin in Europe	7.9	92.1	10.8	6.0	NS	2.6	27.8	***

*P P* value, denotes differences between genders, municipality groups, professions and clinical settings in each statement, *NS* not significant

*P < 0.05, **P < 0.01, ***P < 0.001

^a^The percentages denote respondents who answered ‘any knowledge’, as in any of the response alternatives ‘very little knowledge’, ‘some knowledge’ or ‘good knowledge’

^b^Number of respondents and variations within each column who had responded sufficiently and were included in the statistics

Sixty-two respondents had, despite reported lack of basic knowledge (statements A–D), rated very little to good knowledge according to the statements E and F (see Table 2), commonly followed by comments of logical thinking in the open-answer space. When the 62 respondents were excluded, the total results of *‘‘any knowledge’’* were E = 16.0% (n = 368), and F 19.0% (n = 366).

Respondents with basic knowledge according to peak > 1 were further analysed regarding their sources of knowledge. Sixty-nine of the 72 respondents with peak > 1 specified their sources of knowledge (Table 3). The majority of respondents with self-rated knowledge reported having gained knowledge from colleagues or co-workers, followed by direct clinical experience. The order applied to responses in both municipality groups and among personnel in dental care. For personnel in health care, literature or media, and direct clinical experience, were the most common sources of knowledge. Of the 30 respondents who had gained knowledge through direct clinical experience of IDE, 26 had done it in Sweden, of whom 25 worked in dental care. The other four had gained knowledge through clinical experience abroad.

**Table 3:** Respondents’ sources of knowledge of infant dental enucleation (IDE)

		Direct clinical experience^a^ (%)	Education^b^ (%)	Colleague or co-worker (%)	Literature or media^c^ (%)
Total (n = 69)		43.5	21.8	60.9	29.0
Municipality group					
High (n = 36)		47.2	11.1	55.6	27.8
Low (n = 33)		39.4	33.3	66.7	30.3
Clinical setting					
Health care (n = 16)		25.0	18.8	18.8	56.3
Dental care (n = 53)		49.1	22.6	73.6	20.8

More than one response alternative was allowed

^a^Direct clinical experience: in Sweden or abroad

^b^Education: during undergraduate education, course/seminar after graduation, or other congress/conference

^c^Literature or media: professional/trade journals/magazines, scientific journals or textbooks, other journals, TV

### Clinical experiences of IDE

Of all respondents, 51 (11.7%) did not answer any of the questions about experiences. These worked mainly in health care (dental care 5.6%, health care 12.9%).

Of the remaining 385 respondents, 312 were regularly encountering or had encountered patients below the age of 19 years in their profession. Overall, 39 of these 312 respondents (12.5%) reported having direct clinical experience of at least one patient whom they assessed, immediately or in retrospect, as having been subjected to IDE. Direct clinical experience was more common among dental care personnel than among personnel in health care (36.8 vs. 4.7%; *P* < 0.001). No significant differences were seen in personnel’s direct clinical experiences between municipality groups (high 16.3% vs. low 10.1%; *P* = 0.105). Two female dental hygienists, one from each municipality group, reported that they had ‘been asked by a guardian of or someone else related to an infant, to remove dental buds in an infant’.

Clinical complications assessed as having been caused by IDE had been observed by 22 respondents (20 working in dental care; 13 in the high municipality group). Complications specified were most commonly hypoplasia in or malformation of primary or permanent teeth, reported by 17 respondents, followed by missing primary or permanent teeth, reported by 6 respondents; space- and eruption-related complications, reported by 3 respondents; and others reported by 2 respondents (bone loss, periapical lesion). The hypoplasia in, malformation of or absence of teeth concerned mainly canines.

### Attitudes towards IDE

**Fig. 1:**
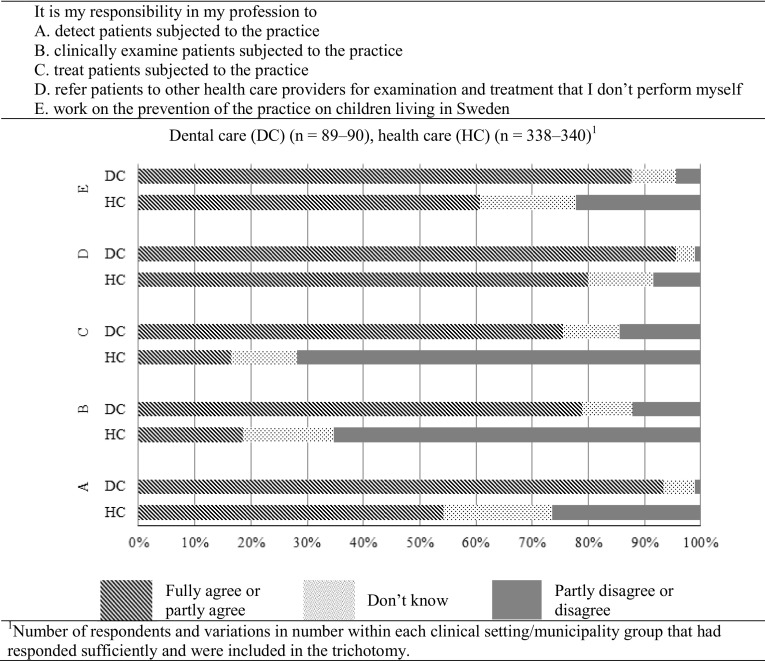
Attitudes among personnel working in dental and health care regarding responsibilities concerning infant dental enucleation (IDE), according to rate of agreement, not knowing or disagreement with each statement

An overview of respondents’ attitudes regarding responsibility for managing IDE is shown in Fig. 1, regarding detection, examination, necessary treatment and referral of performed cases, and prevention of new cases in their clinical settings. More personnel in dental care than in health care fully or partly agreed with having responsibility to detect, examine, treat and refer cases of IDE, and also to work with prevention of new cases of IDE.

Association was analysed between agreeing to having responsibilities regarding detection, examination, treatment, referral and prevention as dependent variables, and working in dental care vs. health care as independent variable. The association is presented in Table 4 as unadjusted and also as adjusted for the independent variables age, work experience in the profession, municipality group, having self-rated basic knowledge according to peak > 1 and having direct clinical experience of IDE. The analysis shows that personnel working in dental were more likely than personnel in health care to fully or partly agree with the statements on responsibility in their settings for detection, examination, treatment, referral and prevention of IDE.

**Table 4:** Association between attitude regarding responsibilities by full or partial agreement and clinical setting—dental care vs. health care

Statements	Unadjusted			Adjusted^a^		
	OR	CI (95%)	*P value*	OR	CI (95%)	*P value*
It is my responsibility in my profession to						
A. detect patients subjected to the practice	11.87	5.05–27.92	< 0.001	8.36	3.22–21.73	< 0.001
B. clinically examine patients subjected to the practice	16.43	9.24–29.21	< 0.001	10.83	5.51–21.31	< 0.001
C. treat patients subjected to the practice	15.68	8.96–27.43	< 0.001	9.65	4.92–18.90	< 0.001
D. refer patients to other practitioners for examination and treatment that I don’t do myself	5.40	1.91–15.22	< 0.001	3.57	1.10–11.59	< 0.05
E. work with prevention of the practice on children living in Sweden	4.60	2.36–8.97	< 0.001	4.49	1.95–10.32	< 0.001

*OR* odds ratio, *CI* confidence interval

^a^Independent variables age (year), working experience in profession (year), municipality group (high vs. low), self-rated basic knowledge (peak > 1), and having direct clinical experience of IDE

## Discussion

This study focused on self-rated knowledge of, clinical experiences of and attitudes towards IDE among dental and health care personnel in 10 Swedish municipalities. The results showed that fewer than one-fifth of all respondents rated themselves as having some degree of basic knowledge of IDE. Approximately a tenth of respondents who had encountered children in their professions reported having seen at least one case that they assessed, immediately or in retrospect, as having been subjected to IDE. Additionally, differences and uncertainty in attitude were seen mainly between clinical settings regarding responsibilities for management and prevention of the practice in their professions.

The general low self-rating of knowledge may reflect a lack of initiatives aimed at a general increase of awareness of the practice within dental and health care. There is, however, a clear difference in self-rated knowledge between personnel in dental care and in health care. This may be due to the nature of the professions, as dentists and dental hygienists routinely inspect and examine oral cavities of their patients during check-ups or treatments. Consequently, there may be a higher possibility of detecting anomalies in oral status, such as missing teeth and manifested complications due to IDE. The results of clinical experiences may support this, as respondents in dental care accounted for the large majority of those with clinical experiences of IDE, with no significant difference between municipality groups. Also, the complications ascribed to the practice by the respondents are comparable to those previously reported in clinical studies (Matee and van Palenstein Helderman 1991; Holan and Mamber 1994; Rodd and Davidson 2000).

The respondents’ sources of knowledge may further contribute to the explanation of difference in self-rated knowledge. Colleagues and/or co-workers, and direct clinical experience, were the first and second most common sources of knowledge, respectively. Information was less commonly obtained through educational programmes and self-education via scientific literature. This indicates that sharing clinical experiences or consulting/discussing unusual findings such as IDE, particularly among dental personnel, may have occurred, leading to some awareness about it. This source, however, may entail a risk that information gained from one’s own direct clinical experience, and/or from co-workers or colleagues, is insufficient or partly incorrect. Nonetheless, the result that knowledge was more than twice as likely to be reported in the high municipality group may also be explained partly by a higher possibility of encountering patients who have had IDE performed as well as learning through shared clinical experiences.

In comparison to dental care personnel, the low self-rating of knowledge among personnel in health care may raise more concerns. The present study sample was not representative of all personnel. However, as we are not aware of initiatives concerning knowledge of the practice, there is little reason for us to assume that knowledge of IDE was significantly higher in other Swedish municipalities. Therefore, the overall self-rating of knowledge points to a need for increased attention in both dental and health care.

The results on attitudes towards responsibilities also show a clear difference between dental and health care personnel. It is reasonable that these differences were greatest regarding responsibility for clinical examinations and treatments. These are primarily areas of expertise for dental personnel. On the other hand, although a majority of respondents did partially or fully agree on detection, referral and prevention being their professional responsibility, a considerable deal of uncertainty and partial to full disagreement was also expressed, mainly by health care personnel. The big difference between dental and health care may well be explained by views of the practice being foremost a matter for dental care services.

It is up for debate whether responsibilities regarding detection, referral and prevention of IDE are legally mandated responsibilities of the surveyed dental and health care professions. The respondents are in professions regulated by several Swedish laws and regulations. One law of particular interest is The Health and Medical Services Act (SFS 1982:763). The act stipulates general responsibilities of health care professions within its purview regarding examination, treatment and prevention of diseases and injuries. The stipulation is reiterated in The National Dental Service Act (SFS 1985:125), which is aimed specifically at regulating dental care.

Additionally, other laws may apply regarding requests for dental bud removal in infants. We find it unlikely that the respondents referred to types of enucleation other than IDE within the scope of the questionnaire. Although only two respondents had been asked to perform it, this indicates that some parents or guardians may still be interested in continuing IDE in Sweden. According to The Social Services Act (SFS 2001:453), all dental and health care personnel in Sweden must report actual or suspected abuse or harm towards individuals below the age of 18 year to social authorities.

IDE may cause both acute and chronic complications that need to be examined, treated and/or controlled in follow-up visits. Lack of awareness and knowledge of IDE, and uncertainty regarding responsibilities concerning it, may have consequences for patients subjected to IDE, as detection and necessary treatment may not occur or may be postponed. Also, enucleations may be performed on children born or living in Sweden. Although IDE may be perceived as an effective and necessary treatment among residents of Eastern African origin (unpublished data), the traditional practice of IDE must be seen as abuse and thereby a violation that is covered by the law. The differences and uncertainties about responsibility regarding management and prevention of IDE therefore point to a need for awareness and clarification of responsibilities according to applicable laws. Lack of knowledge about IDE may represent one of the barriers to clarity regarding responsibilities and their implementation in clinical practices. Knowledge of the practice may therefore provide a proper starting point for all personnel regarding awareness and responsibilities. It is also recommended that the subject is approached with sensitivity in the clinical setting to optimise communication with patients/parents and to avoid alienating them (Rodd and Davidson 2000). Interestingly, the majority responded that IDE needs to be highlighted in their clinical settings, and they requested education on it and guidelines for its prevention. This should be encouraged and accommodated. Appropriate approach methods may therefore require culturally sensitive guidelines. Although this study has limitations, it provides new insight into aspects of IDE to be addressed by educators and policymakers in Sweden.

The study has strengths and limitations that need to be considered. One strength of this study is that it is the first survey on self-rated knowledge, clinical experiences and attitudes concerning IDE in Sweden. Also, it included a variety of professions and clinical settings that constitute a substantial part of health and dental care in which personnel may encounter IDE. The overall response rate was as expected, with a variation of 43.7–73.1% between the professions. As the questionnaires were not assigned individual tracking codes, no personal reminders could be given, which may have impacted on the response rate. Furthermore, no in-depth analysis could be made on non-respondents. Some subdivisions in emergency departments reported that they did not directly manage cases involving children; consequently, some personnel in such settings may have refrained from responding due to a perceived lack of relevance. Additionally, there is a risk of recall bias in questions on knowledge and experience, as respondents may under- or overestimate them (Streiner and Norman 2008), we attempted to limit this risk by having respondents rate their knowledge regarding specific statements on IDE rather than making a general self-assessment of knowledge about it. Ideally, questionnaires may need to be tested for each profession. It was assessed that the respondents were well educated, and the risk for misunderstanding of the questions should be low. Also, as the respondents received basic knowledge through the questionnaire, the subject was introduced to most respondents. Therefore, test–retest may not have fulfilled its purpose.

In conclusion, there was a general low self-rating of knowledge about IDE. There were also considerable differences and uncertainty in attitudes regarding management of IDE. These results suggest a need for increased knowledge of IDE and for clarification of legally mandated responsibilities in dental and health professions. The clarification of responsibilities may be necessary regarding both management of IDE-subjected cases and also the prevention of new cases. Although this study has limitations, it motivates educational initiatives aimed at highlighting the practice and clarifying the responsibilities of dental and health care professionals.
